# A new model defines the minimal set of polymorphism in HLA-DQ and -DR that determines susceptibility and resistance to autoimmune diabetes

**DOI:** 10.1186/1745-6150-3-42

**Published:** 2008-10-14

**Authors:** Christian S Parry, Bernard R Brooks

**Affiliations:** 1 Computational Biophysics Section, Laboratory of Computational Biology, National Heart, Lung, and Blood Institute, National Institutes of Health, Bethesda, Maryland 20892-9314, USA

## Abstract

**Background:**

The mechanism underlying autoimmune diabetes has been difficult to define. There is a strong genetic contribution and numerous studies associate the major histocompatibility complex, especially the class II region, with predisposition or resistance. However, how these molecules are implicated remains obscure.

**Presentation of the hypothesis:**

We have supplemented structural analysis with computational biophysical and sequence analyses and propose an heuristic for distinguishing between human leukocyte antigen molecules that predispose to insulin dependent diabetes mellitus and those that are protective. Polar residues at both β37 and β9 suffice to distinguish accurately between class II alleles that predispose to type 1 diabetes and those that do not. The electrostatic potential within the peptide binding pocket exerts a strong influence on diabetogenic epitopes with basic residues. Diabetes susceptibility alleles are predicted to bind autoantigens strongly with tight affinity, prolonged association and altered cytokine expression profile. Protective alleles bind moderately, and neutral alleles poorly or not at all. Non-Asp β57 is a modifier that supplements disease risk but only in the presence of the polymorphic, polar pair at β9 and β37. The nature of β37 determines resistance on one hand, and susceptibility or dominant protection on the other.

**Conclusion:**

The proposed ideas are illustrated with structural, functional and population studies from the literature. The hypothesis, in turn, rationalizes their results. A plausible mechanism of immune mediated diabetes based on binding affinity and peptide kinetics is discussed. The number of the polymorphic markers present correlates with onset of disease and severity. The molecular elucidation of disease susceptibility and resistance paves the way for risk prediction, treatment and prevention of disease based on analogue peptides.

**Reviewers:**

This article was reviewed by Eugene V. Koonin, Michael Lenardo, Hossam Ashour, and Bhagirath Singh. For the full reviews, please go to the Reviewers' comments section.

## Background

Insulin dependent diabetes mellitus, juvenile diabetes or type 1 diabetes is an inheritable and chronic T cell mediated autoimmune disease. T cells attack and destroy beta cells in the pancreas [OMIM 222100]. The origin or mechanism for this self-directed destruction of insulin producing beta cells has remained elusive and there is no cure. Mechanisms at the basis of type 2 diabetes are understood even less. Type 1 diabetes (T1D) is the most common form of diabetes in children and young people. Its distribution varies by population from 0.1 in 100,000 in China and Venezuela to about 37 in 100,000 in Sardinia and Finland. Two recent reviews give an overview of current understanding, promise and challenge [1,2].

Symptoms accompanying T1D include high levels of glucose in the patient's blood and urine, T cell infiltration into the pancreas and the presence of autoantibodies against insulin and beta cell antigens. The antibodies are under T cell control. Adoptive transfer experiments in animal models confirm that both CD4+ and CD8+ T cells play the dominant role in beta cell destruction [3,4]. B lymphocytes are also important in the pathogenesis of T1D [5]. Beside making antibody markers for beta cell destruction, B cells function as an important subset of antigen presenting cells that maintain the efficient expansion of helper T cells trained on self antigens, as shown in mice [6].

T1D accounts for 5–10% of the incidence of diabetes in the United States but has much more severe and debilitating physical effect on the lives of those affected: secondary effects include renal disease, eye disease leading to blindness, nerve damage, heart and blood vessel disease that may lead to amputation, stroke and premature death. Incidence of T1D is increasing worldwide and there is a need for better molecular and mechanistic understanding to enable better treatment and cure.

There is a strong genetic component to T1D. The clearest association is with the major histocompatibility complex (MHC) locus on chromosome 6p21, especially the class II region. DRA-DRB1*0301 (DR3) and -DRB1*0401 (DR4) are directly implicated as susceptibility molecules [7]. DR3, DR4 or both is present in about 95% of Caucasian patients [8,9], over twice the distribution of these human leukocyte antigen (HLA) alleles in the general population. DQA1*0501-DQB1*0201 (DQ2) and DQA1*0301-DQB1*0302 (DQ8) are considered another set of high risk alleles. Some alleles on the other hand are either negatively associated or confer protection. DRB1*1501 (DR15 or DR2b) and DQA1*0102-DQB1*0602 (DQ0602 or DQ6.2) confer strong dominant protection, even when in combination with alleles with high risk [10]. The apparent distinction between alleles that predispose their carriers to and those that offer protection in T1D incriminates HLA molecules themselves as causative factors. All or almost all autoimmune diseases associate with the MHC [11]. Nevertheless, not all carriers of the high risk class II variants develop the disease. Current opinion is that autoimmune diabetes results from the complex interaction between many genes and the environment with MHC class II region as a major influencing factor.

The prevailing idea that links the HLA with autoimmune diabetes is the *β57 hypothesis *[12]. *β57 hypothesis *implicates the association of mutations of the strongly conserved aspartic acid at position 57 of the class II β-chain in HLA-DQ, to Ala, Val or Ser, with susceptibility to disease. Crystallographic structures show that the conserved salt bridge between β57 Asp and the invariant α76 Arg in class II MHC is broken when β57 Asp is mutated [13-15]. This mutation has been interpreted to render open the C-terminus of the peptide binding groove, to reduced stability of class II MHC molecules and to promiscuous peptide binding [16,17]. DQA1*0301-DQB1*0302 (DQ8) and DQA1*0501-DQB1*0201 (DQ2), both presumed to predispose to T1D, lack β57 Asp. Similar arguments have been made for the mouse non obese diabetes (NOD) model I-Ag7 molecule considered the equivalent of the human DQ8 [18,19]. But *β57 hypothesis *fails to explain the strong association of DR3 and DR4. DR3 and DR4 retain β57 Asp. While non-Asp β57 alleles are increased in Caucasian T1D patients [12] other population studies show overall high prevalence of the conserved β57 Asp in HLA alleles of diabetics [20-22]. DR and DQ loci are in tight linkage disequilibrium and this makes it difficult to delineate the relative contributions of DR and DQ. It was therefore debated whether DR is also a predisposing locus [23]. As is now accepted, both DR and DQ contribute to a neutral response, susceptibility or dominant protection in T1D [24,25]. DRB1, with DR3 and DR4, has been shown to be a major susceptibility factor [25,26]. Non-Asp β57 fails to explain the molecular basis of T1D. The thrust of current research has shifted to genome-wide association studies.

The promise of structural biology is to elucidate such structure-function relationship and disease association. A number of crystallographic structures of class II MHC molecules associated with autoimmune diseases have been determined: HLA-DR2 [27,28], -DR3 [29], -DR4 [30], -DQ2 [31], -DQ6 [32], -DQ8 [15], the mouse I-Ak [33] and I-Ag7 [13,14]. Notwithstanding, the molecular connection to disease or resistance remains obscure [34-36]. Our recently reported structure of DRA-DRB3*0101 (DR52a) [37] has kindled our interest in HLA association with autoimmune diseases. DR52a has the replacement β57 Asp to Val, a structural similarity to HLA-DQ molecules associated with susceptibility. But DR52a is not an at risk allele with respect to T1D. In the same way, DR7 has non-Asp β57 and is not associated with T1D. Because of public health and economic consequences there is interest in understanding the molecular basis of autoimmune diabetes for screening and risk prediction that may lead to early therapeutic intervention.

Using insight from experimental structure determination and the evolutionary relationship between class II HLA molecules [37], we have carried out detailed biophysical and sequence analyses of class II molecules and interpreted the results in the context of published functional and epidemiological studies the question of HLA link with T1D. We have identified for the first time and propose that the physical chemical nature of the polymorphic residues at β9 and β37 determine class II HLA susceptibility, resistance or dominant protection in T1D.

## Presentation of the hypothesis

**Figure 1:**
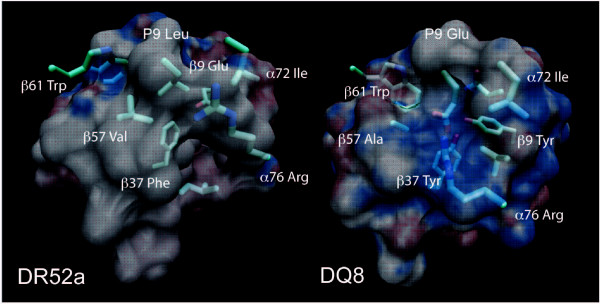
**Electrostatic potential map of the P9 pocket**. Electrostatic potential maps have been calculated separately for DR52a and DQ8 without their peptide and mapped to their van der Waals surface. The P9 pockets are shown here with their respective peptide P9 anchor side chain and selected residues in the pocket superimposed. The DR52a pocket is neutral and DQ8 shows a highly positive (blue) potential. The difference reflects the preference for a hydrophobic peptide side chain in DR52a P9 versus the preference for an acidic side chain in DQ8. The electrostatic difference is hypothesized to be a factor in susceptibility to T1D. Details of the calculations are given under supplementary materials (Additional file 1).

We hypothesized and confirmed that differences in polymorphic residues in pocket P9 of DR3 and DR52a, closely related molecules, lead to differences in the charge character and epitope selection (not shown). DR3 and DR52a have overlapping but distinct peptide specificity. While DR3 is strongly associated with T1D, DR52a is not. The significant electrostatic difference between DR3 and DR52a is in their P9 pockets [37]. Following this lead we compared the respective electrostatic surfaces of DR52a [37] and DQ8 [15]. DQ8 is strongly associated with T1D. Electrostatic potentials of the two molecules (without peptide) were obtained by solving the nonlinear Poisson-Boltzmann equation (additional file 1) and mapped to their respective *van der Waals *surface (Figure 1).

DR52a surface electrostatic potential in pocket P9 is neutral. In clear contrast, the diabetes disposing DQ8 has high positive potential. We therefore postulate that the difference at P9 ensues from polarizable polymorphic residues: β9 Glu (in DR52a) versus Tyr (in DQ8) and β37 Phe (in DR52a) versus Tyr (in DQ8). In the structures, mutation of the strongly conserved β57 Asp breaks the salt bridge between β57 and the invariant α76 Arg [15,37]. In DR52a, α76 Arg swings upward and away from pocket P9. In DQ8 α76 Arg maintains the same lateral axis as the salt bridge but bends sharply inward toward the peptide P9 residue Glu. β37 in DR52a is hydrophobic (Phe). This abolishes a crucial hydrogen bond between an acidic peptide residue and pocket 9. Therefore DR52a has no preference for a peptide acidic residue at P9 [37]. β37 in DQ8 is Tyr. The difference in orientation of α76 Arg in the two structures is determined by the nature of β37.

**Figure 2:**
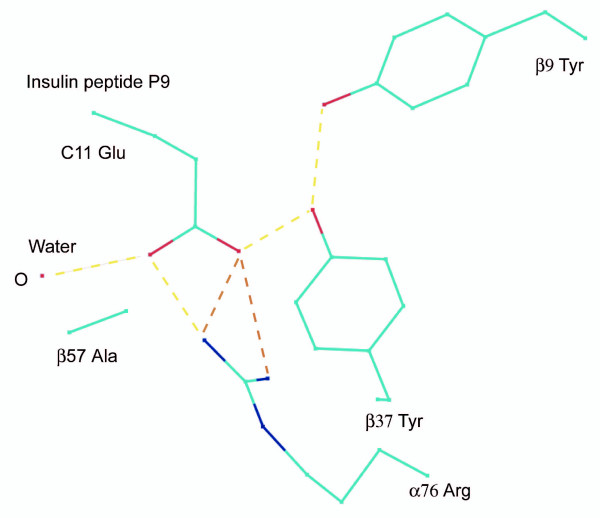
**Hydrogen bond interactions involving insulin B chain P9 anchor in DQ8**. There is a hydrogen bonding network involving the insulin peptide glutamic acid anchor and DQ8 α76 arginine, crystallographic water, β37 tyrosine, and β9 tyrosine. Two of the bonds from α76 are shown with relaxed constraints. This network of bonds hyper-stabilizes the insulin peptide.

When the peptides are superimposed over their respective mapped surface, we are struck by the multiple hydrogen bonds and coupled interactions between DQ8 and insulin chain B: 9–23 SHLVEALYLVCGERG (P1, P4 and P9 anchors underlined). A network of bonds involving α76 Arg, β37 Tyr, tethered water and β9 Tyr from DQ8 impose a firm grip on the insulin B peptide at P9 (Figures 1 and 2). β37 Tyr makes a short hydrogen bond to the peptide P9 residue carboxylate atom (2.73 Å). These unique interactions are hypothesized to result in unusually strong binding of the peptide and slower dissociation kinetics leading to an altered cytokine expression profile. Cytokine expression and CD4+ T cell helper response depend on peptide-class II MHC-affinity. Limited changes in the residues of the peptide or in the MHC binding site alter the T cell response: to non-responsiveness, partial response leading to "anergy" and to differences in proliferation and cytokine response [38,39].

Next, we examine to what extent these structural features found in DQ8 but not in DR52a persist in class II alleles associated with autoimmune diabetes. We correlate T1D susceptibility with the electrostatic potential via the three polymorphic markers: β9, β37 and β57. The heuristic uses a simple count of polar or charged residues in HLA pocket P9 as score, surrogate for an exact calculation of the electrostatic potential or count of potential hydrogen bonds in the absence of atomic coordinates. We compared the results using epidemiological and genotype analyses. Table 1 lists major class II HLA alleles, whether they possess any of these three markers and whether they are associated with T1D according to published literature [references in Table S1 (Additional file 2)].

**Table 1:** The three-residue grip model of susceptibility to immune mediated diabetes

allele	β9	β37	Non-Asp β57	score	T1D
DRB1*0101	Hydrophobic W	Polar S	No	1	No
DRB1*0301	Polar E	Polar N	No	2	Yes
DRB1*0401	Polar E	Polar Y	No	2	Yes
DRB1*0402	Polar E	Polar Y	No	2	Yes
DRB1*0403	Polar E	Polar Y	No	2	No
DRB1*0404	Polar E	Polar Y	No	2	Yes
DRB1*0405	Polar E	Polar Y	Yes S	3	Yes
DRB1*0406	Polar E	Polar S	No	2	No
DRB1*0407	Polar E	Polar Y	No	2	Yes
DRB1*0408	Polar E	Polar E	No	2	Yes
DRB1*0409	Polar E	Polar Y	Yes S	3	Yes
DRB1*07	Hydrophobic W	Hydrophobic F	Yes V	1	No
DRB1*0801	Polar E	Polar Y	Yes S	3	Yes
DRB1*0901	Charged K	Polar N	Yes V	3	Yes
DRB1*1001	Polar E	Polar Y	No	2	No
DRB1*11	Polar E	Polar Y/N/S/D	No	2	Yes
DRB1*11	Polar E	Hydrophobic, F	No	1	No
DRB1*12	Polar E	Hydrophobic L/F	Yes V	2	No
DRB1*1301	Polar E	Polar N	No	2	Yes
DRB1*1303	Polar E	Polar Y	Yes S	3	Yes
DRB1*1401	Polar E	Hydrophobic F	Yes A	2	No
DRB1*1402	Polar E	Polar N	No	2	No
DRB1*15	Hydrophobic W	Polar S	No	1	No
DRB1*16	Hydrophobic W	Polar S	No	1	No
DRB3*0101	Polar E	Hydrophobic F	Yes V	2	No
DRB3*0200	Polar E	Polar Y	No	2	Yes
DRB5*0202	Polar Q	Polar D/N	No	2	Yes
DQB1*05	Polar Y	Polar Y	Yes V	3	Yes
DQB1*0201	Polar Y	Hydrophobic I	Yes A	2	Yes
DQB1*0301	Polar Y	Polar Y	No	2	Yes
DQB1*0302	Polar Y	Polar Y	Yes A	3	Yes
DQB1*0303	Polar Y	Polar Y	No	2	Yes
DQB1*0601	Hydrophobic L	Polar D	No	1	No
DQB1*0602	Hydrophobic F	Polar Y	No	1	No
DQB1*0603	Polar Y	Polar Y	No	2	No
DQB1*0604	Polar Y	Polar Y	Yes V	3	Yes

Polymorphic residues within pocket P9 of class II MHC alleles are scored according to whether they are polar/charged (+1), hydrophobic (0) or whether the conserved β57 aspartic acid is replaced (+1). A replacement at β57 Asp leaves α76 Arg free to coordinate an acidic peptide anchor in P9, or when the pocket is hydrophobic move out of the pocket. Two polar residues in the pocket or a polar residue and β57 aspartic acid mutation distinguishes between alleles that predispose to diabetes and those that do not with few exceptions. Diabetes association in column 6 is culled from the published literature. DR11 is listed both as a susceptibility allele and as a resistant (neutral) allele. Typing was to two significant figures; this obscures the nature of residue at β37.

Excellent correlation is observed between the presence of these markers and predisposition to or protection from autoimmune diabetes. Table 1 shows that every allele possessing all three polar markers does predispose to T1D without exception. Secondly, in most cases alleles with two positive markers predispose to T1D. Conversely, in no instance does a class II HLA allele without or with only one of these three markers associate with T1D. This simple criterion suggests that HLA susceptibility to T1D is a function of residual charge or polarizable residues in the P9 pocket of the class II molecule.

The presence of two of three markers β9 (polar), β37 (polar) and β57 (non-Asp), score of "2" (Table 1), distinguishes between predisposing and non-predisposing molecules with remarkable accuracy. This is the general rule. Well known high risk alleles DRB1*0301, DRB1*0401 and DRB1*1301 score at "2" due to the presence of polar residues at β9 and β37. Other alleles strongly predisposing to T1D, DRB1*0405, DRB1*0409, DRB1*0901, DQ*0302, or DQB1*0604, have a score of "3." They possess the two polar residues as well as replacement of the strongly conserved β57 Asp. Mouse I-Ag7 also has a score of "3" in this scheme. The haplotype associated with the highest risk DR3/DR4-DQ8 is consistent with this analysis. On the other hand, DR7 (score of "1" due to β57 Asp to Val mutation; β9 and β37 hydrophobic) is not predisposing. The protective alleles DRB1*1501 and DQB*0602 have scores of "1" due to a polar residue at β37. Using the strict criterion of "2" the model fails to predict in only a few instances.

The same data represented in terms of the three components β9, β37 and β57 is even more instructive (Table 2). The rules governing HLA association with T1D may be stated clearly as:

**Table 2:** Clustering of HLA alleles in the space defined by the polar polymorphic residues in P9

β9	β37	β57	Alleles and association with type 1 diabetes
Polar	Polar	non-Asp	DRB1*0405, DRB1*0409, DRB1*0801, DRB1*0901, DRB1*1303, DQB1*05, DQB1*0302, DQB1*0604

Polar	Polar	Asp	DRB1*0301, DRB1*0401, DRB1*0402, DRB1*0403, DRB1*0404, DRB1*0406, DRB1*0407, DRB1*0408, DQB1*0301, DQB1*0303, DQB1*0603, DRB5*0200, DRB3*0202, DRB1*1301, DRB1*11, DRB1*10, DRB1*1402

Polar	Hydrophobic	non-Asp	DQB1*0201, DRB3*0101, DRB1*12

Polar	Hydrophobic	Asp	DRB1*1401, DRB1*11

Hydrophobic	Polar	non-Asp	none

Hydrophobic	Polar	Asp	DQB1*0601, DQB1*0602, DRB1*1501, DRB1*16, DRB1*0101

Hydrophobic	Hydrophobic	non-Asp	DRB1*07

Hydrophobic	Hydrophobic	Asp	none

HLA alleles are placed in the table based on whether the markers β9 and β37 are polar or hydrophobic and whether or not they retain the strongly conserved aspartic acid at β57. Susceptible molecules are shown in **bold**, neutral in normal text, and those that confer protection are shown in *italics*. All HLA molecules susceptible to autoimmune diabetes possess polar residues at β9 and β37. The combination of β9 and β37 suffice to describe susceptibility to type 1 diabetes, active protection or resistance (neutral). Non-Asp β57 is a modifier. DRB1*0406 has the short Ser at β37. DRB1*0403 may be neutral or protective. DRB1*0101 also has Ser at β37 which is not effective in conferring dominant protection. This analysis does not find DQ2 (DQB1*0201) to be high risk and it is proposed to be non-disposing. Disease association is taken from the literature as in Table 1 [Table S1 (Additional file 2)].

1. Polar residues at both β9 and β37 accurately distinguish between predisposing and non-predisposing class II HLA molecules.

2. A single polar residue at β37 but not at β9 confers dominant protection.

3. A hydrophobic residue at β37 offers passive resistance (neutral).

Two polar residues at β9 and β37 are proposed as the least common structural feature, the minimal set of polymorphism, in DQ and DR molecules that predispose to T1D. Non-Asp β57 is not a risk factor by itself. Non-Asp β57 is a modifier or additional factor that increases disease risk in the presence of the polar pair. These three residues represent a useful set of markers for predicting HLA association with T1D, disease risk and severity.

## Implications of the hypothesis

This hypothesis employs simple physical ideas: residual charge in a pocket, peptide-class II MHC binding affinity, kinetics and competition are used as parameters in susceptibility or resistance to T1D. Alleles that confer susceptibility to disease are predicted to bind diabetogenic peptides strongly with slow dissociation kinetics. Protective alleles are predicted to bind less tightly but able to compete with susceptibility alleles for diabetogenic peptides. Molecules that are neutral or resistant bind poorly or not at all.

Such tight binding susceptibility alleles have been reported, DRB5*0101 [40,41] and DQ8 [15]. These, on average, present a kinetically homogenous and more organized complex. There is little entropic cost in binding such pre-organized complexes by T cells. These need receptors of only low to moderate affinity and are likely to draw a broad range of T cell clones of diverse specificity. A broad range of T cell clones elicited for diabetes predisposing alleles have the potential to cross-react.

The peptide in the moderate binding protective allele such as DQB1*0602, on the other hand, is predicted to have a faster dissociating rate. The peptide-protective allele complex is more heterogeneous in conformation and rich in entropy. Moderately binding peptide-MHC complexes are predicted to select only a narrow range of finely tuned, rearranged T cell receptors of high affinity. In the ensuing clonal dynamics, the protective T cell clones, with higher affinity receptors, thrive against T cells with restriction on susceptibility alleles. Peptide-MHC affinity influences T cell proliferation, clonal expansion and deletion.

This model offers an alternative by using difference in affinity in place of "immunodominance" and "tolerance." Basic processes such as *van der Waals *interactions, hydrogen bond formation, covalent bonds, electrostatic interactions of dipoles and stereochemical complementarity suffice to explain most biological phenomena [42]. We have used the biophysical analysis of the properties of peptide-class II MHC binding to tease out the "diabetogenic mutations" underlying T1D and propose similar analysis to uncover the etiological polymorphism underlying class HLA involvement in other autoimmune diseases such as multiple sclerosis, myasthenia gravis, Graves disease, Addison's disease and celiac disease.

### Susceptibility to disease

The model suggests that increased affinity of diabetes autoantigens to MHC molecules and hyperstable binding are crucial variables underlying T1D. Insulin B: 9–23 peptide P9 anchor in the DQ8 structure lies in a strong electrostatic field and held tightly by coupled nonbonded interactions (Figures 1 and 2). β37 Tyr coordinates the peptide P9 Glu in a critical hydrogen bond. Tyrosine hydrogen bonds are strong [43]. β37 Tyr exerts a strong pull on a diabetogenic peptide acidic, polar or basic residue. This is predicted to disrupt normal on-off kinetics. The insulin B peptide will therefore have a very large kinetic half life. A salt bridge formed between α76 Arg and the peptide P9 Glu is similarly strong. Insulin, an important self antigen in the pathogenesis of T1D is also expressed in the thymus. In "thymic education" of immature T cells, or the activation of autoreactive T cells in pancreatic draining lymph nodes and in islets, such prolonged confrontation, from the long half-life of autoantigenic peptides with class II molecules, could be a sustaining factor. This could elicit an inflammatory response and subsequent attack by killer T cells and self directed antibodies. High affinity binding and slow dissociation rates arising from a network of nonbonded interactions at P9 involving β9, β37, and α76 Arg (non-Asp β57) are postulated to underlie HLA association with T1D. The threshold for class II MHC association is the presence of a polar pair of residues at β9 and β37. This synthesis also holds for other T1D predisposing epitopes from insulin, GAD65, IA-2 and other peptides from islet beta cells with acidic or polar residues at P9.

### Diabetes epitopes and motifs

Many diabetogenic epitopes when aligned show a high incidence of acidic residues at their C-termini. Epitopes of DQ8 show a preponderance of acidic residues at P1 and P9, about 39% and 60% incidence, respectively, with typically a hydrophobic residue at P4; NOD I-Ag7 displays a similar motif [44]. Well characterized peptides, GAD65 207–220 TNMFTYEIAPVFVLLEYVT, insulin B 9–23 SHLVEALYLVCGERG, islet cell antigen (ICA69) 33–50 TKQAFIKATGKKEDEHVV, and IA-2 961–979 FEFALTAVAEEVNAIL show this pattern (Table 3). Peptides from GAD65 and insulin bind in the same frame to both DQ8 and mouse I-Ag7 [15,44,45] pointing to similarities in their binding sites. Many of these epitopes are also presented by DR3, DR4 and DR2a (DRB5*0101) [46]. Each shows a preference for polar – usually acidic – residues at P9. Like DQ8 and NOD I-Ag-7, these high risk alleles possess polar residues at both β9 and 37. By comparison, class II MHC alleles such as DR7 and DR52a that are neutral or resistant to T1D show no such preference for acidic or polar residues at P9 (Table 3). DR7 and DR52a possess a hydrophobic residue at β37.

**Table 3:** Selected alleles, their representative peptides and motifs

Peptide	Sequence and motif	allele
	**1**xx**4**x**6**xx**9**	
CLIP peptide 81–104	LPKPPKPVSK**M**RM**A**T**P**LL**M**QALPM	DR3
MET proto-oncogene 724–735	**L**KI**D**L**A**NR**E**TSI	DR3
HSP70 M leprae 261–280	DSDKNP**L**FL**D**E**Q**LI**R**AEFQR	DR3
HSP70 M leprae 408–427	QPS**V**QI**Q**V**Y**QG**E**REIASHNK	DR3
SSX2 37–51	WEK**M**KA**S**E**K**IF**Y**VYM	DR3
Tetanus toxin 830–843	QY**I**KA**N**S**K**FI**G**ITE	DR3
Preproinsulin 78–88	QP**L**AL**E**G**S**LQ**K**	DR4
ER-60 protease	TEDE**F**KK**F**I**S**DK**D**ASVVG	DR4
14-3-3 epsilon 57–71	RAS**W**RI**I**S**S**IE**Q**KEE	DR4
GAD65 274–286	IA**F**TS**E**H**S**HF**S**LK	DR4
GAD65 471–490	VDKCLELAE**Y**LY**N**I**I**KN**R**EG	DR4
Aminopeptidase 462–475	**F**EL**F**P**S**LS**H**NLLVD	DR4
β2 microglobulin 64–78	L**Y**YT**E**F**T**PT**E**KDEY	DR0405
der pII 58–73	IDG**L**EV**D**V**P**GI**D**PNA	DR0405
HSP90 beta 68–81	KELK**I**DI**I**P**N**PQ**E**R	DR0405
Fel d1 22–37	EQVAQ**Y**KA**L**P**V**VL**E**NA	DR0405
Integrin β3 24–39	A**W**CS**D**E**A**LP**L**GSPRCD	DR52a
AChR alpha 144–163	MKLGT**W**TY**D**G**S**VV**A**INPESD	DR52a
AChR epsilon 201–219	ENGE**W**AI**D**F**C**PG**V**IRRHHG	DR52a
Lol p1 101–120	AP**Y**HF**D**L**S**GH**A**FGSMAKKG	DR52a
Influenza A HA 306–324	PK**Y**VK**Q**N**T**LK**L**ATGMRNVP	DR7
HSP70 M Leprae 241–260	AKIE**L**SS**S**Q**S**TS**V**NLPYITV	DR7
HIV-1 RT 326–345	**F**RK**Q**N**P**DI**V**IQYMDDLYVG	DR7
HLA class I α52–60	**I**EQ**E**G**P**EY**W**	DQ2
Flu NP 265–278	I**A**SN**E**N**M**DA**M**ESSTL	DQ2
M leprae 18 kD 31–43	D**A**WR**E**G**E**EF**V**VEF	DQ2
Rabies virus NP 11–24	NNQ**V**VS**L**K**P**EI**I**VDQ	DQ2
Thyroid peroxidase 632–645	IDV**W**LG**G**L**A**EN**F**LP	DQ2
Thyroid peroxidase 632–645	I**D**VW**L**G**G**LA**E**NFLP	DQ8
Trail receptor 2 364–380	GRFT**Y**QN**A**A**A**QP**E**TG	DQ8
Cyclophilin R 325–340	VDQW**S**TE**T**I**A**SH**E**DIE	DQ8
Cathepsin D 65–77	EPV**S**EL**L**K**N**YL**D**A	DQ8
E25B protein 112–126	YQTI**E**EN**I**K**I**FE**E**DA	DQ8
I-A2B 644–658	GGDPG**A**DA**T**A**A**YQ**E**L	DQ8
I-A2B 762–776	KN**R**SL**A**V**L**TY**D**HSRV	DQ8
GAD65 121–140	YVVKS**F**DR**S**T**K**VI**D**FHYPNE	DQ8
GAD65 231–250	PGGSGDGI**F**SP**G**G**A**IS**N**MYA	DQ8
GAD65 248–259	N**Y**AM**M**I**A**RF**K**MF	DR3
GAD65 250–273	AMMIAR**F**KM**F**P**E**VK**E**KGMAALPRL	DQ8
GAD65 471–490	VDKCLELA**EYLYNIIKN**REG	DR4
I-A2 961–979	FE**F**AL**T**A**V**AE**E**VNAIL	DQ8
I-A2 961–979	F**E**FA**L**T**A**VA**E**EVNAIL	DQ8
ICA69 33–50	TKQA**F**IK**A**T**G**KK**E**DEHVV	DQ8
Insulin B:9–23	SHLV**E**AL**Y**L**V**CG**E**RG	DQ8
Insulin B:24-C:4	F**F**YT**P**K**T**RR**E**AED	DQ8

Epitopes of several class II MHC molecules are aligned manually according to their pocket motif. The major diabetes susceptibility molecules DR3, DR4. DR405 and DQ8 show a strong preference for polar residues at position P9. Acidic residues such as Asp and Glu are common at this position but there are also basic and other polar residues. In contrast alleles that are neutral (DR7 and DR52a) show a preference for aliphatic and hydrophobic residues at P9. DQ2 shows no preference for acidic residues at P9 that characterize diabetogenic molecules. Sequence information comes from several sources including [46].

Zinc transporter protein, ZnT8, is a newly identified autoantigen [47]. T1D predisposing epitopes from ZnT8 have not been defined yet. Nevertheless, consistent with its role as a cation transporter, the sequence is rich with acidic, basic or polar residues, especially in the terminal regions. Potential epitopes sharing the common motif may be derived from this.

### Protection

Table 2 shows that protective alleles segregate at a score of "1": DR15 (DRB1*15), DR16 (DRB1*16) and DQ6 (DQB1*0602). A score of "1" may be interpreted as intermediate or normal binding affinity for an insulin or a similar diabetogenic peptide. Protective alleles possess a single polar residue at β37 able to interact directly with a polar or acidic peptide residue at P9. Given a limiting concentration of peptides in the cellular compartment, molecules that confer dominant protection can compete successfully with susceptibility alleles. Clones of T cells trained on protective alleles are hypothesized to compete successfully and bring about the contraction of clones restricted on T1D disposing class II alleles in clonal competition.

### Resistance or neutrality to disease

DRB3*0101 does not bind to T1D associated peptides. In the structure of DR52a, β57 Val abolishes the salt bridge at the rim of the binding site and releases α76 Arg to a marked conformation up and away from pocket 9. β37 Phe coupled with Val at β57 strongly determines the extreme α76 Arg conformation in the PlA1-DRB3*0101 complex (PDB code 2Q6W). These combine to make a very hydrophobic P9 pocket and little preference for charged or polar peptide residues at P9. The peptide binding motif (P1, P4, P9) of DR52a is [**hydrophobic **– polar – **hydrophobic**] compared to the motif for the diabetes associated HLA-DQ8 and the mouse counterpart I-Ag7, [polar – **hydrophobic **– polar]. DR52a possesses two of the markers, polar β9 and non-Asp β57. However, β37 Phe determines the conformation of α76 Arg. β37 Phe also breaks the hydrogen bonding network characteristic of diabetogenic alleles. A similar argument can be made for DR7 and for DRB1*1401, DRB1*12 and a subset of alleles of DRB1*11. All possess a hydrophobic residue at β37. In the framework of our competition model, class II molecules such as DR7, DR52a, DR12, DR14 and alleles of DR11 with hydrophobic residue at β37 show no preference for peptides with acidic P9. They do not bind and cannot compete for such diabetogenic peptides. They also cannot offer dominant protection but are resistant (neutral) to autoimmune diabetes. A hydrophobic residue at β37 renders a class II allele neutral to T1D.

### The determinant role of β37

The physical chemical nature of β37 is consequential to genetic susceptibility, resistance and protection. A hydrophobic residue at β37 acts as stopper and resists predisposition to autoimmune diabetes. Interestingly, β37 and β57 are correlated such that a hydrophobic residue at β37 ensures a hydrophobic residue at β57; else β57 encodes the conserved Asp (Table 4) which forms a restraining salt bridge to α76 Arg. Therefore a hydrophobic residue at β37 imparts a strong hydrophobic character to pocket P9. A hydrophobic pocket P9 has no preference for acidic residues that characterize diabetogenic epitopes. The hypothesized role of β37 may be discerned in a comparison of DRB3 alleles: while DR52a (DRB3*0101; β37 Phe) is neutral to T1D, DR52b (DRB3*0200) is predisposing to autoimmune diabetes in Belgian and in Northern Indian populations [48,49]. DR52b (DRB3*02) alleles encode a polar group at β37, usually Tyr and occasionally Asn or Ser, hence a preference for a basic residue in pocket P9. These are similar to the predisposing DR3.

**Table 4:** Pattern of variation between β37 and β57 in class II MHC when β37 is hydrophobic

Class II MHC molecule	β37	β57
DRB1*0701	F	V

DRB1*0706	F	A

DRB1*0708	V	V

DRB1*0709	F	V

DRB1*0809	F	D

DRB1*0821	F	D

DRB1*1110	F	D

DRB1*1201	L	V

DRB1*1204	L	D

DRB1*1205	L	V

DRB1*1308	L	D

DRB1*1364	L	D

DRB1*1401	F	A

DRB1*1405	F	D

DRB1*1407	F	A

DRB3*0101	F	V

DRB3*0301	F	V

DQB1*0201	I	A

DQB1*0203	I	D

In class II MHC molecules, a hydrophobic residue at β37 ensures a hydrophobic residue at β57 or else retains the strongly conserved aspartic acid. In the latter case a salt bridge is maintained between β57 Asp and the invariant α76 Arg which is thereby "latched." A hydrophobic β37 when coupled with a replacement of β57 makes the P9 pocket hydrophobic and tends to move α76 Arg out of the pocket as in DR52a or in DQ2. This mollifies diabetes association and explains the neutral association of DRB1*12 and DRB3*0101. Hydrophobic β37 is proposed as the basis of resistance to type 1 diabetes.

The nature of the polar residue at β37 is also important for dominant protection. This may be discerned in the case of DRB1*0406 which has a score of "2" but is protective, in apparent disagreement with the hypothesis. DRB1*0404, on the other hand, with a score of "2" is predisposing. The protective DRB1*0406 possesses the short serine which is not as effective as the long tyrosine in DRB1*0404 in maintaining the hydrogen bond network that connects the diabetes associated peptide residue. β37 Ser, nevertheless, renders pocket P9 polar. Further, in the DR2 haplotype, DQB1*0602 shows greater dominant protection than DRB1*1501. Both are scored "1." But while DQB1*0602 encodes β37 Tyr, DRB1*1501 has β37 Ser. β37 tyrosine in DQB1*0602 is more effective in making a putative single bond to the peptide acidic P9, is able to compete better against potential predisposing alleles and thus offers stronger protection. The presence of a single polar residue at β37 (hydrophobic β9) capable of making a strong interaction with an acidic or polar peptide residue in P9 offers strong protection. Incidentally, there is no instance of hydrophobic β9, polar β37 and non-Asp β57 (Table 2). A plausible explanation is that this combination has been selected against in MHC evolutionary history in favor of this protective role for β37.

A major implication of this hypothesis is that DQ2 is not deemed a high risk or predisposing allele (Table 2). The risk commonly accorded to DQ2 derives from bona fide high risk alleles carried in the haplotype, DR3 or DQ8. Like DR52a, a hydrophobic β37 in DQ2 renders the pocket P9 hydrophobic and no particular preference for negatively charged peptide anchor residues characteristic of diabetogenic peptides [37,50]. DQ2 has isoleucine and alanine at β37 and β57, respectively.

The analysis explains a major shortcoming of the β57 hypothesis, the observation that the DR7-DQ2 haplotype (DRB1*0701 – DQA1*0201 DQB1*0202) does not predispose to T1D [12] though both DR7 and DQB1*0202 have non-Asp β57. Both DR7 and DQ2, however, encode a hydrophobic β37, which explains the observation. In comparison, the DR3-DQ2 haplotype predisposes to T1D. DR3 possesses polar residues at both β9 and β37; the risk is imparted by DR3. This also supports the study of Shtauvere and colleagues who found that it is DR3 – and not DQ2 – that is the susceptibility molecule [51], and rationalizes the contrarian results of Kockum et al., [52] who found DQ2 to be the more 'protective' allele with DR3 the susceptibility factor in the DR3-DQ2 combination, and consistent with the findings of Krokowski and coworkers [26]. DQ2 carries the DQA1*0501 α-chain. With respect to the difference in DQ αchains, Johansen and coworkers have shown that the α-chain has little effect on epitope selection of DQ2 [53]. Altogether, hydrophobic β37 is a mollifying influence on β57 replacement. A hydrophobic β37 breaks the hydrogen bond network that involves β9 and α76 Arg that are hypothesized to place a firm grip on the peptide at P9 (Figure 2).

### The role of β9

The role of β9 in this analysis is illustrated in DQB1*06 alleles in the transition from DQB1*0602 to *0603. DQB1*0603 is not as protective as DQB1*0602 but not predisposing to disease as *0604. In the DR2 haplotype, DRB1*1501 (DR2b; β9 hydrophobic Trp, score of "1") is protective while DR2a (DRB5; β9 polar, score of "2"; both β9 and β37 polar) is predisposing to T1D. While DRB5 is expressed with the protective DRB1*15/16 the protection tendered by β37 Ser (in DRB1*15/16) is not as dominant as with β37 Tyr in DQB1*0602.

### β57 in context

The three node scheme readily accommodates the old β*57 hypothesis *[12]. Interpreted through our model, the strongly conserved β57 Asp acts as a latch that restrains α76 Arg through the conserved salt bridge. Non-Asp β57 releases α76 Arg for a potential bond to the carboxylate group in a T1D associated peptide but only when both residues at β37 and β9 are polar; this increases the hold on the peptide.

### Severity and onset of disease

There is a correlation between the number of positive markers and susceptibility to disease. The gradation poses the question, to what extent the number of polar markers, surrogates for electrostatics, determine severity or early onset of clinical symptoms. The hypothesis predicts a correlation between the number of the three positive markers in the haplotype and early onset and severity of disease. The high risk haplotype DRB1*0401 DQB1*0302 has many of the interactions described while the protective DRB1*1501 DQB1*0602 has few of these, and the neutral DRB1*0701 DQB1*0201 is unable to form such interactions because of the hydrophobic residue at β37. DRB1*0901 (DR9) possesses all three markers and a very polar P9 pocket. DRB1*0901 has the strongly polar lysine at β9. This places a buried partial charge within pocket P9. DRB1*0901 is unique to Asians. Likewise, the susceptibility allele DRB1*0405 has all three positive markers (β9 Glu, β37 Tyr and β57 Ser). These are associated with rapid onset and severity of disease. Like DRB1*0901 and DRB1*0405, DQ9, DQB1*0604 and DQB1*0401 are prevalent in Japan. We suspect that the interactions elucidated here play a role in the novel fulminant type 1 diabetes reported in Japan [54].

### Specific cases

DRB1*1001, DRB1*11 (DR11) and DRB1*0403 are exceptions to the "2" threshold criterion. For DRB1*1001 and DRB1*0403, it is possible that something other than these three markers influences features of peptide binding that are not captured in this model, for example, the mode of binding of the peptide, or are influenced by other molecules in the haplotype, or other pockets. With DR11, the literature is conflicting as the genotype does not resolve different suballeles. Both polar (Tyr, Asn, Asp and Ser) and hydrophobic (Phe) residues are encoded at β37 in DR11.

DQ4 (DQA1*0301-DQB1*0401) possesses β57 Asp yet is a high risk allele. It encodes tyrosine at β37 and phenylalanine at β9. With a score of "1" it should be protective. But DQ4 has the long leucine at β56. This poses steric hindrance in the P9 pocket that would push the α-chain away leaving α76 Arg unpaired with β57 Asp and free to interact with the peptide P9 residue, as in DQ8. Leucine at β56 is rare and appears only in DQB1*0401 and DQB1*0402. Unlatching α76 Arg gives DQ4 an effective score of "2" (polar Tyr at β37 plus freed α76 Arg). This explanation is corroborated by our observation that in the sequence of the isomorphous HLA-DP molecules there is a preponderance of another long residue, glutamic acid, at β56. Interestingly, in this case the conserved residue at β57 is glutamic acid (instead of aspartic acid in DQ and DR) apparently to compensate in length for the expected salt bridge with the invariant DP α76 Arg.

### I-Ag7 and weak binding peptides

**Figure 3:**
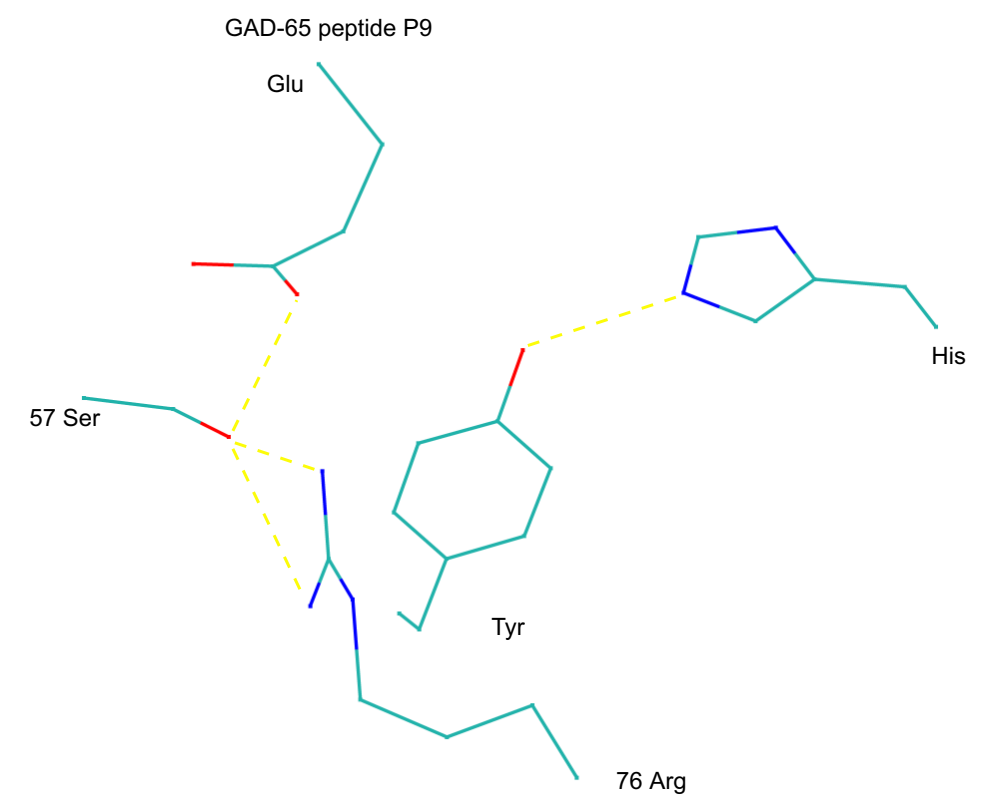
**Hydrogen bond interactions involving GAD65 P9 Glu in I-Ag7**. Hydrogen bonding interactions between the peptide P9 glutamic acid and residues in mouse I-Ag7 are shown. There is notably no hydrogen bond between the peptide P9 Glu and β37 Tyr. There is instead an important hydrogen bond between peptide P9 Glu and β57 Ser. A hydrogen bond is also maintained between α76 Arg and β57 Ser. These interactions vary from the equivalent interactions between insulin B peptide P9 Glu in human DQ8.

The NOD mouse is a valuable experimental model for autoimmune diabetes; it possesses I-Ag7 as the sole class II molecule [55]. I-Ag7 bears non-Asp β57, a structural feature shared by DQ8. I-Ag7 and DQ8 have similar specificity for selected peptides [44] (Table 3). It has been demonstrated that weak binding peptides with fast dissociation times characterize dominant epitopes of I-Ag7. A biological role is proposed for weak binding peptides in escape from tolerance leading to autoimmunity [56,57]. Insulin chain B 9–23 is a dominant epitope common to both mouse I-Ag7 and human DQ8. Insulin B 9–23 binds I-Ag7 weakly (3–10 umol/l) with a relatively short dissociation constant (< 2 hours). These observations are examined:

The shared structural characteristics between I-Ag7 and DQ8 include polar β9 (His in I-Ag7 and Tyr in DQ8) and polar β37 (Tyr in both I-Ag7 and DQ8) in addition to non-Asp β57 (Ser in I-Ag7 and Ala in DQ8). The rare histidine at β9 in I-Ag7 is charged and constrains the reactive phenolic hydroxyl group of β37 Tyr through a strong hydrogen bond of 2.92 Å, from the crystal structure of I-Ag7 with bound HEL peptide (PDB code 1F3J; [14]). The strong constraint on β37 Tyr by β9 His is retained in the structure of I-Ag7 with GAD65 epitope which has negatively charged Glu at P9 (PDB code 1ES0; [13]) (bond distance His NE2-Tyr OH = 2.86 Å). The structural constraint on β37 Tyr due to charged β9 His results in a change of rotamer in the tyrosine that prevents hydrogen bond formation between β37 Tyr and the acidic residue from the peptide (Figure 3). Histidine is notably shorter than tyrosine. Therefore the histidine pulls tyrosine at β37. This effects subtle but important structural changes in pocket 9 of I-Ag7. While polar β37 Tyr and β9 His lend a polar nature to pocket P9, β37 Tyr is not able to exert the strong, direct pull on the acidic peptide residue at P9, as in DQ8. β9 His constraint on β37 Tyr is also conserved (bond distance His NE2-Tyr OH = 2.64 Å) in the structure of mouse I-Ak (PDB code 1IAK; [33]).

The stereochemical rearrangement involving β37 Tyr may also explain the multiple preference at pocket P9 of I-Ag7 for acidic as well as hydrophobic residues and the dual register for insulin B 9–23 [44]. Pocket 9 in mouse I-Ag7 is therefore more permissive as observed in a comparison of the peptide repertoire of I-Ag7 and DQ8. By contrast, in DQ8 a strong hydrogen bond is formed between β37 Tyr and the peptide Glu (Figure 2). The multiplicity of bonds between the insulin peptide P9 Glu and DQ8 pocket 9 is noteworthy. Measured affinity of DQ8 with insulin B 9–23 is ~nM and the dissociation constant > 72 hours [58] versus ~uM and dissociation constant < 2 hours in I-Ag7 [56]. The inability of I-Ag7 to form a similar dense network of bonds with the peptide negatively charged residue likely accounts for the weak peptide binding and fast dissociation times reported. It is noteworthy that histidine is never encoded at β9 in human DQ and DR molecules. Subtle but significant structural differences in pocket 9 due to β9 and β37 determine the difference in reactivity of the two molecules. This calls for caution in extending mouse I-Ag7 binding data to human class II molecules.

It may well be that weak binding dominant peptides escape from tolerance in the thymus, as observed in I-Ag7 [56]. The counter example of strong binding of the same insulin B epitope to DQ8 argues against this as a general rule. It has also been demonstrated that weak binding peptides to I-Ag7 can induce tolerance [59]. Other experiments show that for either weak binding or tight binding epitopes, altered or competing ligands are a means of achieving tolerance. T cells specific for the altered and competing peptides may successfully compete against T cells that recognize dominant (or self) peptides, and thus such altered peptides may be capable of "tolerizing" diabetogenic determinants. Seen in this respect weak binding or competing peptides may be used as a strategy of developing therapies against T1D and other autoimmune diseases.

## Testing the hypothesis

We have shown that residual charge in pocket 9 of class II MHC DQ and DR underlies the strong linkage between the HLA and autoimmune diabetes. The hypotheses presented above are testable by peptide binding analyses and by animal models. The binding affinity of diabetogenic epitopes containing acidic residues to diabetes associated class II molecules and those not associated (neutral or protective) may be measured and compared. Similarly, corresponding peptide association and dissociation kinetics may be compared. In another test using the mouse NOD model, β37 tyrosine in the lone class II molecule I-Ag7 may be replaced by phenylalanine, another hydrophobic residue or serine. Serine is polar but short. In another experiment a transgenic mouse incorporating just HLA-DQ2 could be used to test the role of β37 and whether DQ2 confers susceptibility or resistance to T1D.

Peptides of moderate affinity are suggested as analogues for use as competitor against diabetes associated epitopes in class II MHC binding as a means of preventing the autoimmune response. The optimal parameters for analogue peptides may be deduced from peptide-MHC and T cell assays. Such experiments will also be useful to determine how the relative affinity and concentration of an analogue peptide can be used to modulate the binding equilibrium, cytokine expression profile and T helper phenotype appropriately for treatment.

## Competing interests

The authors declare that they have no competing interests.

## Authors' contributions

CP conceived and developed the hypothesis, carried out the analysis, wrote and edited the manuscript. BB discussed the analysis and results, and supported the project. Both authors have read and approved the final manuscript.

## Reviewers' comments

### Reviewer report 1

Dr. Eugene Koonin, National Center for Biotechnology Information, National Library of Medicine, National Institutes of Health, Bethesda, Maryland.

This is a very clear and straightforward analysis of type I diabetes-associated polymorphisms in class II HLA molecules that leads to the delineation of a minimal set of diabetes-associated markers. Notably, this minimal set consists of just three amino acid positions, and Parry and Brooks find a convincing mechanistic explanation in the charge changes in the peptide-binding pocket P9. The hypothesis is readily testable in straightforward peptide-binding experiments.

#### Author response

We are grateful to Dr. Koonin for his review. Your other suggestions have also improved this article. We have a restructured and more streamlined manuscript. The paper is much shorter.

### Reviewer report 2

Dr. Michael Lenardo and Dr. Hossam Ashour, Molecular Development Section, Laboratory of Immunology, National Institute of Allergy and Infectious Diseases, National Institutes of Health, Bethesda, Maryland.

The manuscript by Parry and Brooks addresses polymorphisms in HLA-DQ and HLA-DR as potential modifiers of susceptibility and resistance in Type 1 Diabetes (T1D). They employed structural, biophysical and computational approaches and relied heavily on published literature to propose T1D susceptibility and protective HLA-antigen molecules. The manuscript is generally informative, with several instances of ambiguity, as outlined below in my report.

Major Comments:

1 – The authors focused their efforts on Insulin and did not address other autoantigenic peptides that were shown to be major players in T1D pathogenesis such as GAD, IA-2, Zn transporter, and HSP. What is the reason for that?

2 – Table S1, Are there any controversies in the literature regarding a particular allele being associated with T1D? The authors can denote the references that are not definitive (due to conflicting reports from other investigators in the field), if any, and discuss this further in the supplemental section.

#### Author response

We would like to thank Drs. Lenardo and Ashour very much for their comments above. They have helped to make our paper stronger and more precise. Our approach is structural but broadly bioinformatic and we have used the published literature to test how general our structural/biophysical hypothesis is.

1. The revised manuscript includes a new section "Diabetes epitopes and motifs" and an additional table, Table 3. These address and expand the hypothesis to other autoantigens. We are particularly thankful for alerting us to the role of the zinc transporter.

2. We have addressed ambiguity in the literature concerning whether alleles predispose to diseases, are neutral or protective (Table S1). This ambiguity arises mostly from low resolution genotyping.

### Reviewers

We have read the revised manuscript and find your changes to have strengthened and clarify the work. We believe that it is now ready for publication.

#### Reviewer report 3

Dr. Bhagirath Singh, Institute of Infection and Immunity, Canadian Institutes of Health Research (CIHR), and Department of Microbiology & Immunology, University of Western Ontario, London, Ontario, CANADA.

This manuscript presents a hypothesis that suggests that a minimal set of polymorphism in HLA-DQ and -DR specifies susceptibility and resistance to autoimmune diabetes. The authors have done a detailed analysis of the structural data of various class II human major histocompatibility (MHC) molecules and the hypothesis extends the current understanding of the linkage between class II MHC and type 1 diabetes (T1D). The pioneering work of McDevitt et al [12] showed the role of beta chain Asp-57 residue of certain HLA molecules in autoimmune diabetes. The present paper extends this to include beta chain residues 9, 37 and 57. The analysis presented is logical and supports the revised and extended hypothesis. I have number of suggestions to improve the presentation and interpretations presented by the authors:

1. The title and the abstract should be modified to give a better understanding of the contents of the manuscript. I would suggest a modified title:

"A new model defines the minimal set of polymorphism in HLA-DQ and -DR that determines susceptibility and resistance to autoimmune diabetes" The abstract should include new elements that extends and improves the current model of the role of MHC class II in autoimmune diabetes. It should mention the role of peptide affinity for MHC class II and its influence on T cell selection as discussed on page 20–21 *(in submitted manuscript)*. The proposed studies that may test the model could also be mentioned.

2. Taking into account the published structure-function studies with diabetes relevant epitopes of GAD and Insulin the authors should explain if their model (involving beta chain residues 9, 37 and 57) supports the binding to MHC class II or functional activation of T cells with various peptide analogs. The studies of Insulin B 9–23 or various GAD peptides binding to I-Ag7 or DQ8 will be highly relevant.

3. It is not clear on p. 21 *(in submitted manuscript)* what is meant by " in the model proposed by this report the protective molecule is predicated to bind less tightly but competitively". There are recent studies (Suri A. et al Curr Opin Immunol. 2008; 20:105–10) that showed that low affinity peptides for MHC class II binding could induce diabetes. These studies are relevant in making the point that weak affinity of autoimmune epitopes for MHC class II may control autoreactive T cell responses. The high to medium affinity peptide specific T cells are probably deleted in the thymic selection. Thus, how low affinity of peptide for MHC class II may relate autoreactive T cell selection may be important point to be discussed in the manuscript.

#### Author response

1. We embrace the suggestion of Dr. Singh and adopt the new title. We have also expanded the abstract.

2. In the model, class II MHC molecules that predispose to autoimmune diabetes, For example, DQ8 binds (very) tightly to diabetogenic epitopes while a protective alleles such as DQ6.2 binds moderately (ie. with intermediate affinity). We propose using analogues that can compete for binding to the class II molecule. We anticipate that such peptides need to bind only moderately but with sufficient affinity to compete for the class II MHC binding site. These would interact in an equivalent manner with beta chain residues β9 and β37.

A simple strategy we envision is inspired by DRB1*0406. DRB1*0406 possesses polar residues at both β9 and β37 except that β37 is Ser. The long Glu at P9 in the epitopes listed in Table 3 ay be changed to Ser to make simple altered ligands. In the text, we discuss how such altered ligands are bound by class II molecules and activate T cells. Two references we cite by Chaturvedi et al. and by Evavold at al. [38,39] give excellent discussions about such functional activation of T cells by peptide analogs. More sophisticated strategies may also be used to make peptide mimics that successfully compete with diabetogenic peptides, especially in alleles with non-Asp β57, to defeat the coordination by α76 Arg. We are very thankful. This comment helps to expand the scope of the paper.

A new section "Diabetes epitopes and motifs" and the additional Table 3 address the role of GAD and other autoantigens, and illustrate the properties of ligands that bind HLA alleles associated with T1D.

3. You raise a very important point. The idea that weak binding epitopes, rather than high affinity peptides, underlie type 1 diabetes (Suri et al., Curr Op Immunol, 2008 [56]) appears to run counter to our hypothesis. First, we try to explain this observation of weak binding peptides in the mouse I-Ag7 at the level of structure. The new section "I-Ag7 and weak binding peptides" is devoted to this question.

But now we also see how weak affinity peptides may be used to control autoimmunity as you suggest: we propose analogue peptides which though weak binding may be able to shift the equilibrium from the tight binding complexes of diabetogenic epitopes and susceptibility alleles to complexes between analogue peptides and susceptibility alleles. This strategy needs to take into account the affinity or the relative affinity of the analogue peptide as well as its concentration. These will have to be determined empirically. How the two parameters influence the cytokine profile is of interest.

Again, we are thankful for this important suggestion.

#### Reviewer

The manuscript has been considerably revised and updated based upon my comments. Additional sections have been added and new references provided. In my view, the revised manuscript provides a new model to test the hypothesis that in addition to residue 57, residues 9 and 37 on the beta chain provide additional sites on MHC class II molecule for peptide presentation to CD4+ T cells. In association with Table 1 and 2, the list of peptides and motifs in Table 3 provides a good correlation in structure-function. The presentation of autoantigenic peptides by MHC class II molecules in susceptible host determines their ability to induce protective or pathogenic T cells in autoimmune diseases. A better understanding of the structure-function relationship of MHC class II molecules is critical in the rational design of relevant molecules to prevent autoimmune diseases.

## Supplementary Material

Additional file 1 Parameters for nonlinear Poisson-Boltzmann calculations. Click here for file

Additional file 2 HLA association with type 1 diabetes and incidence across populations according to the published literature. Click here for file
