# Supplementary material for: A new model defines the minimal set of polymorphism in HLA-DQ and -DR that determines susceptibility and resistance to autoimmune diabetes
Source: Biol Direct. 2008 Oct 14;3:42. doi: 10.1186/1745-6150-3-42 (PMC2590596; doi:10.1186/1745-6150-3-42)
Supplement: Additional file 1 — Parameters for nonlinear Poisson-Boltzmann calculations. [file 1745-6150-3-42-S1.doc]

## Additional file 1 - Parameters for nonlinear Poisson-Boltzmann calculations

Coordinates for DRA-DRB3*0101 (DR52a) (PDB code 2Q6W) [1] and DQA1*0301-DQB1*0302 (DQ8) (PDB code 1JK8) [2] were obtained from NCBI <http://www.ncbi.nlm.nih.gov/Structure/>. Missing hydrogen atoms were added and the atomic coordinates were modified to include atomic charge and radius parameters using values from the Charmm force field (Charmm27) [3]. We evaluated the electrostatic distribution in DR52a and DQ8, respectively, without peptide. The electrostatic potential ϕ(x) incorporates detailed information of the shape of the structure and charge distribution, and was obtained as a solution to the nonlinear Poisson-Boltzmann partial differential equation in a grid based finite difference scheme in solvent media:

, x ∈ Ω [4].

Protein partial charges were approximated as fixed charges and modeled as delta functions of magnitude [q_i_] centered at [x_i_] (right hand side of the equation). The mobile counterion charges are modeled in a continuous “mean field approximation” (implicit solvent) that incorporates the charges, bulk concentration, the steric potential and solvent accessibility (second term on the left hand side). ε(x) is the position dependent dielectric function and κ^2^ contains solvent accessibility information; both incorporate atomic positions and radii. The equation is solved in the protein domain Ω subject to some fixed potential g(x), the Dirichlet boundary condition. Parameters used are given in the table below.

The calculations were repeated in vacuum as reference.

Table. Parameters for nonlinear Poisson-Boltzmann calculations in APBS [4] for HLA-DR52a and DQ8

| Parameter | DR52a | DQ8 |
| --- | --- | --- |
| Grid dimension | 161 193 161 (in Å) | 161 129 193 |
| Coarse grid length | 105.21 131.61 105.48 (in Å) | 114.49 96.76 135.04 |
| Fine grid length | 81.89 97.42 82.04 (in Å) | 87.35 76.92 99.43 |
| Grid spacing | 0.33 Å | 0.33 |
| Protein dielectric | 2.0 | 2.0 |
| Solvent dielectric | 78.54 | 78.54 |
| Solvent radius | 1.40 Å | 1.40 |
| Solvent density | 10 | 10 |
| Surface tension | 0.105 kJ/mol/Å^2^ | 0.105 |
| Temperature | 298.15 K | 298.15 |

**Supplementary references**

1. Parry CS, Gorski J, Stern LJ: **Crystallographic structure of the human leukocyte antigen DRA, DRB3*0101: models of a directional alloimmune response and autoimmunity.** *J Mol Biol* 2007, **371**:435-446.

2. Lee KH, Wucherpfennig KW, Wiley DC: **Structure of a human insulin peptide-HLA-DQ8 complex and susceptibility to type 1 diabetes.** *Nat Immunol* 2001, **2**:501-507.

3. Brooks BR, Bruccoleri RE, Olafson BD, States DJ, Swaminathan S, Karplus M: **CHARMM: a program for macromolecular energy, minimization and dynamics calculations.** *J Comput Chem* 1983, **4**:187-217.

4. Baker NA: **Poisson-Boltzmann methods for biomolecular electrostatics.** *Methods Enzymol* 2004, **383**:94-118.
