# Supplementary material for: A new model defines the minimal set of polymorphism in HLA-DQ and -DR that determines susceptibility and resistance to autoimmune diabetes
Source: Biol Direct. 2008 Oct 14;3:42. doi: 10.1186/1745-6150-3-42 (PMC2590596; doi:10.1186/1745-6150-3-42)
Supplement: Additional file 2 — HLA association with type 1 diabetes and incidence across populations according to the published literature. [file 1745-6150-3-42-S2.doc]

**Supplementary Table S1** Representative HLA diabetes association studies from the published literature.

| **HLA allele** | **Reference** |
| --- | --- |
| DRB1*01 | [1-8] |
| DRB1*03 | [5, 9-12] |
| DRB1*0301 | [1, 4, 6-8, 13-18] |
| DRB1*04 | [1, 2, 5-7, 9, 10, 12, 16-18] |
| DRB1*0401 | [4, 7, 8, 10, 11, 13, 15, 16] |
| DRB1*0402 | [7, 8, 13] |
| DRB1*0403 | [7, 8, 12-14, 19] |
| DRB1*0404 | [7, 8, 12, 13, 16] |
| DRB1*0405 | [7, 8, 12, 14, 16, 19] |
| DRB1*0406 | [14] |
| DRB1*0407 | [7, 14] |
| DRB1*0408 | [7, 11] |
| DRB1*07 | [7, 8, 16, 20, 21] |
| DRB1*08 | [1, 7, 8, 22, 23] |
| DRB1*0802 | [19] |
| DRB1*09 | [7, 17, 24] |
| DRB1*0901 | [7, 14, 19] |
| DRB1*10 | [7, 14] |
| DRB1*11 | [7, 12, 21] |
| DRB1*12 | [7, 14, 16] |
| DRB1*13 | [5-7, 21] |
| DRB1*1301 | [25] |
| DRB1*1302 | [2] |
| DRB1*14 | [7, 14] |
| DRB1*15 | [5, 7, 14] |
| DRB1*1501 | [2, 4, 8, 9, 12, 26, 27] |
| DRB1*1503 | [11] |
| DRB1*16 | [7, 28] |
| DRB3*0101 | [20] |
| DRB3*0200 | [29, 30] |
| DRB5 | [28, 31] |
| DRB5*0101 | [2, 15, 26, 27] |
| DQ2 | [5, 7-9, 12, 16] |
| DQB*0201 | [8, 9, 12, 14, 15, 32] |
| DQ7 | [5] |
| DQB*0301 | [7, 12, 14, 16, 28, 33] |
| DQ8 | [5, 11, 12, 34] |
| DQB*0302 | [7, 9, 10, 12-14, 16, 32] |
| DQB*0303 | [7, 8, 19, 24, 33, 35] |
| DQB*0401 | [7, 10, 14, 19] |
| DQB*0402 | [7, 11, 12, 23, 33] |
| DQ5 | [5] |
| DQB1*05 | [7, 16, 27, 33, 36] |
| DQB*06 | [5, 7, 11] |
| DQB*0601 | [7, 14] |
| DQB*0602 | [7, 9, 12, 14, 27, 32] |
| DQB*0603 | [7, 32, 36] |
| DQB*0604 | [7, 32, 33] |

The literature is sometimes conflicting. One reason is the varying degree of resolution in gene typing. As resolution to four places is not always available this may obscure the nature of the residue at a critical positions. In other instances, unreliable typing by serology is reported. DR5 and DR11 are such examples. Another reason is the nature of population studies; these may be limited by small sample size. All of the above may contribute to discrepancies. Not all reports resolve DQB1*0301 from 0302. There appears to be discrepancy in DR1 association with T1D. Park et al., [14] find DR1 mildly predisposing while majority of reports find it not predisposing. In this case, sample size could influence the results, or the statistics may not be significant. This could also arise from the associated molecules in the haplotype, such as DRB1*0407, even though the well known culprits DR3 and DR9 were removed. Similarly, discrepancy in reported association of DRB5*01 (DR2a) could result from DRB5*0101 susceptibility masked by the protective effects of DRB1*1501 DQB1*0602 within the DR2 haplotype.

References [7] and [8] are recent sources of alleles and their association with type 1 diabetes.

**Supplementary references**

1. Noble JA, Valdes AM, Cook M, Klitz W, Thomson G, Erlich HA: **The role of HLA class II genes in insulin-dependent diabetes mellitus: molecular analysis of 180 Caucasian, multiplex families.** *Am J Hum Genet* 1996, **59**:1134-1148.

2. Bach JM, Otto H, Nepom GT, Jung G, Cohen H, Timsit J, Boitard C, van Endert PM: **High affinity presentation of an autoantigenic peptide in type I diabetes by an HLA class II protein encoded in a haplotype protecting from disease.** *Journal of Autoimmunity* 1997, **10**:375-386.

3. Bach JM, Otto H, Jung G, Cohen H, Boitard C, Bach JF, van Endert PM: **Identification of mimicry peptides based on sequential motifs of epitopes derived from 65-kDa glutamic acid decarboxylase.** *Eur J Immunol* 1998, **28**:1902-1910.

4. Geluk A, van Meijgaarden KE, Schloot NC, Drijfhout JW, Ottenhoff TH, Roep BO: **HLA-DR binding analysis of peptides from islet antigens in IDDM.** *Diabetes* 1998, **47**:1594-1601.

5. Shtauvere A, Rumba I, Dzivite I, Sanjeevi CB: **HLA-DR and -DQ gene polymorphism in Latvian patients with insulin-dependent diabetes mellitus.** *Tissue Antigens* 1998, **52**:385-388.

6. Bearzatto M, Naserke H, Piquer S, Koczwara K, Lampasona V, Williams A, Christie MR, Bingley PJ, Ziegler AG, Bonifacio E: **Two distinctly HLA-associated contiguous linear epitopes uniquely expressed within the islet antigen 2 molecule are major autoantibody epitopes of the diabetes-specific tyrosine phosphatase-like protein autoantigens.** *J Immunol* 2002, **168**:4202-4208.

7. Thomson G, Valdes AM, Noble JA, Kockum I, Grote MN, Najman J *et al.*: **Relative predispositional effects of HLA class II DRB1-DQB1 haplotypes and genotypes on type 1 diabetes: a meta-analysis.** *Tissue Antigens* 2007, **70**:110-127.

8. Erlich H, Valdes AM, Noble J, Carlson JA, Varney M, Concannon P *et al.*: **HLA DR-DQ haplotypes and genotypes and type 1 diabetes risk: analysis of the type 1 diabetes genetics consortium families.** *Diabetes* 2008, **57**:1084-1092.

9. Leech NJ, Kitabchi AE, Gaur LK, Hagopian WA, Hansen J, Burghen GA, Palmer JP, Nepom GT: **Genetic and immunological markers of insulin dependent diabetes in Black Americans.** *Autoimmunity* 1995, **22**:27-32.

10. Park YS, She JX, Noble JA, Erlich HA, Eisenbarth GS: **Transracial evidence for the influence of the homologous HLA DR-DQ haplotype on transmission of HLA DR4 haplotypes to diabetic children.** *Tissue Antigens* 2001, **57**:185-191.

11. Heward JM, Mijovic CH, Kelly MA, Morrison E, Barnett AH: **HLA-DQ and DRB1 polymorphism and susceptibility to type 1 diabetes in Jamaica.** *Eur J Immunogenet* 2002, **29**:47-52.

12. Abid Kamoun H, Hmida S, Kaabi H, Abid A, Slimane Houissa H, Maamar M *et al.*: **HLA polymorphism in type 1 diabetes Tunisians.** *Ann Genet* 2002, **45**:45-50.

13. Harfouch-Hammoud E, Timsit J, Boitard C, Bach JF, Caillat-Zucman S: **Contribution of DRB1*04 variants to predisposition to or protection from insulin dependent diabetes mellitus is independent of dq.** *J Autoimmun* 1996, **9**:411-414.

14. Park YS, Wang CY, Ko KW, Yang SW, Park M, Yang MC, She JX: **Combinations of HLA DR and DQ molecules determine the susceptibility to insulin-dependent diabetes mellitus in Koreans.** *Hum Immunol* 1998, **59**:794-801.

15. Harfouch-Hammoud E, Walk T, Otto H, Jung G, Bach JF, van Endert PM, Caillat-Zucman S: **Identification of peptides from autoantigens GAD65 and IA-2 that bind to HLA class II molecules predisposing to or protecting from type 1 diabetes.** *Diabetes* 1999, **48**:1937-1947.

16. Petrone A, Bugawan TL, Mesturino CA, Nistico L, Galgani A, Giorgi G, Cascino I, Erlich HA, Di Mario U, Buzzetti R: **The distribution of HLA class II susceptible/protective haplotypes could partially explain the low incidence of type 1 diabetes in continental Italy (Lazio region).** *Tissue Antigens* 2001, **58**:385-394.

17. Chen BH, Chung SB, Chiang W, Chao MC: **GAD65 antibody prevalence and association with thyroid antibodies, HLA-DR in Chinese children with type 1 diabetes mellitus.** *Diabetes Res Clin Pract* 2001, **54**:27-32.

18. Bilbao JR, Calvo B, Aransay AM, Martin-Pagola A, Perez de Nanclares G, Aly TA *et al.*: **Conserved extended haplotypes discriminate HLA-DR3-homozygous Basque patients with type 1 diabetes mellitus and celiac disease.** *Genes Immun* 2006, **7**:550-554.

19. Maruyama T, Shimada A, Kasuga A, Kasatani T, Ozawa Y, Ishii M *et al.*: **Analysis of MHC class II antigens in Japanese IDDM by a novel HLA-typing method, hybridization protection assay.** *Diabetes Res Clin Pract* 1994, **23**:77-84.

20. Todd JA, Bell JI, McDevitt HO: **HLA-DQ beta gene contributes to susceptibility and resistance to insulin-dependent diabetes mellitus.** *Nature* 1987, **329**:599-604.

21. Balducci-Silano PL, Layrisse Z, Dominguez E, Amaro R, Gunczler P, Lanes R, Zaro R: **HLA-DQA1 and DQB1 allele and genotype contribution to IDDM susceptibility in an ethnically mixed population.** *Eur J Immunogenet* 1994, **21**:405-414.

22. Thomson G: **HLA DR antigens and susceptibility to insulin-dependent diabetes mellitus.** *Am J Hum Genet* 1984, **36**:1309-1317.

23. Dubois-Laforgue D, Timsit J, Djilali-Saiah I, Boitard C, Caillat-Zucman S: **Insulin-dependent diabetes mellitus in non-DR3/non-DR4 subjects.** *Hum Immunol* 1997, **57**:104-109.

24. Lee HC, Ikegami H, Fujisawa T, Ogihara T, Park SW, Chung YS *et al.*: **Role of HLA class II alleles in Korean patients with IDDM.** *Diabetes Res Clin Pract* 1996, **31**:9-15.

25. Mbanya JC, Sobngwi E, Mbanya DN: **HLA-DRB1, -DQA1, -DQB1 and DPB1 susceptibility alleles in Cameroonian type 1 diabetes patients and controls.** *Eur J Immunogenet* 2001, **28**:459-462.

26. Vogt AB, Kropshofer H, Kalbacher H, Kalbus M, Rammensee HG, Coligan JE, Martin R: **Ligand motifs of HLA-DRB5*0101 and DRB1*1501 molecules delineated from self-peptides.** *J Immunol* 1994, **153**:1665-1673.

27. Reijonen H, Ilonen J, Akerblom HK, Knip M, Dosch HM: **Multi-locus analysis of HLA class II genes in DR2-positive IDDM haplotypes in Finland. The &quot;Childhood Diabetes in Finland&quot; (DiMe) Study Group.** *Tissue Antigens* 1994, **43**:1-6.

28. Sanjeevi CB, Lybrand TP, Landin-Olsson M, Kockum I, Dahlquist G, Hagopian WA, Palmer JP, Lernmark A: **Analysis of antibody markers, DRB1, DRB5, DQA1 and DQB1 genes and modeling of DR2 molecules in DR2-positive patients with insulin-dependent diabetes mellitus.** *Tissue Antigens* 1994, **44**:110-119.

29. Buyse I, Sandkuyl LA, Zamani Ghabanbasani M, Gu XX, Bouillon R, Bex M *et al.*: **Association of particular HLA class II alleles, haplotypes and genotypes with susceptibility to IDDM in the Belgian population.** *Diabetologia* 1994, **37**:808-817.

30. Mehra NK, Kaur G, Kanga U, Tandon N: **Immunogenetics of autoimmune diseases in Asian Indians.** *Ann N Y Acad Sci* 2002, **958**:333-336.

31. Zeliszewski D, Tiercy JM, Boitard C, Gu XF, Loche M, Krishnamoorthy R *et al.*: **Extensive study of DRB, DQA, and DQB gene polymorphism in 23 DR2-positive, insulin-dependent diabetes mellitus patients.** *Hum Immunol* 1992, **33**:140-147.

32. Sanjeevi CB: **HLA-DQ6-mediated protection in IDDM.** *Hum Immunol* 2000, **61**:148-153.

33. Undlien DE, Kockum I, Ronningen KS, Lowe R, Saanjeevi CB, Graham J, Lie BA, Akselsen HE, Lernmark A, Thorsby E: **HLA associations in type 1 diabetes among patients not carrying high-risk DR3-DQ2 or DR4-DQ8 haplotypes.** *Tissue Antigens* 1999, **54**:543-551.

34. Kelemen K, Gottlieb PA, Putnam AL, Davidson HW, Wegmann DR, Hutton JC: **HLA-DQ8-associated T cell responses to the diabetes autoantigen phogrin (IA-2 beta) in human prediabetes.** *J Immunol* 2004, **172**:3955-3962.

35. Graham J, Kockum I, Sanjeevi CB, Landin-Olsson M, Nystrom L, Sundkvist G *et al.*: **Negative association between type 1 diabetes and HLA DQB1*0602-DQA1*0102 is attenuated with age at onset. Swedish Childhood Diabetes Study Group.** *Eur J Immunogenet* 1999, **26**:117-127.

36. Hermann R, Turpeinen H, Laine AP, Veijola R, Knip M, Simell O, Sipila I, Akerblom HK, Ilonen J: **HLA DR-DQ-encoded genetic determinants of childhood-onset type 1 diabetes in Finland: an analysis of 622 nuclear families.** *Tissue Antigens* 2003, **62**:162-169.
